# 

**Published:** 1908-01-04

**Authors:** 


### BOOKS RECEIVED.

J. AND A. CHURCHILL.

"Diseases of the Eye." By J. Herbert Parsons, D.Sc., M.B., B.S., F.R.C.S.

"Surgical Pathology." By Anthony A. Bowlby, C.M.G., F.R.C.S. 5th edition.

"Materia Medica, Pharmacy, Pharmacology, and Therapeutics." By W. Hale White, M.D., F.R.C.P. 10th edition.

"The Ophthalmoscope." By Gustavus Hartridge, F.R.C.S. 5th edition.

"The Book of Receipts" (Beasley). Rewritten by E. W. Lucas, F.I.C., F.C.S. 11th edition.

"The Operations of Surgery." 5th edition. By W. H. A. Jacobson, M.Ch.Oxon, F.R.C.S., and R. P. Rowlands, M.S.Lond., F.R.C.S.

"The Book of Prescriptions." By E. W. Lucas, F.I.C., F.C.S.

ERNEST BELL.

"The New Ethics." By J. Howard Moore.

JOHN BALE, SONS AND DANIELSSON, LTD.

"Air, Light, and Sun Baths." By Dr. A. Montennis. Translated from the French by Fred. Rothwell.

"Laboratory Studies in Tropical Medicine." Second edition. By C. W. Daniels, M.B., and A. T. Stanton, M.D.

"Some Successful Prescriptions." By A. Herbert Hart, M.D.

"Proceedings of the Conference on the Teaching of Hygiene and Temperance in the Universities and Schools of the British Army."

"Syphilis in the Army." By Major H. C. French, R.A.M.C.

"The Pyronex, its Theory and Practice."

W. H. AND L. COLLINGRIDGE.

"The City Diary, 1908."

T. NELSON AND SONS.

"The Odd Women." By George Gissing. Nelson's Library.

"Pride and Prejudice." By Jane Austen.

"Matthew Austin." By W. E. Norris (Nelson's Library).

### BOOKS AT ONE-THIRD COST.

Thousands of the Best Books at from 25 to 80 per cent. below the original prices. Largest and best Stock of Second-hand and New Remainder Books in the World. Write for our JANUARY Catalogue.

W. H. SMITH & SON,
Library Department, 186 Strand, London, W.C.

